# Role of Virus-Encoded microRNAs in Avian Viral Diseases

**DOI:** 10.3390/v6031379

**Published:** 2014-03-21

**Authors:** Yongxiu Yao, Venugopal Nair

**Affiliations:** Avian Viral Diseases, The Pirbright Institute, Compton Laboratory, Berkshire RG20 7NN, UK; E-Mail: yongxiu.yao@pirbright.ac.uk

**Keywords:** avian viruses, MDV, avian leukosis virus, DEV, ILTV, microRNAs

## Abstract

With total dependence on the host cell, several viruses have adopted strategies to modulate the host cellular environment, including the modulation of microRNA (miRNA) pathway through virus-encoded miRNAs. Several avian viruses, mostly herpesviruses, have been shown to encode a number of novel miRNAs. These include the highly oncogenic Marek’s disease virus-1 (26 miRNAs), avirulent Marek’s disease virus-2 (36 miRNAs), herpesvirus of turkeys (28 miRNAs), infectious laryngotracheitis virus (10 miRNAs), duck enteritis virus (33 miRNAs) and avian leukosis virus (2 miRNAs). Despite the closer antigenic and phylogenetic relationship among some of the herpesviruses, miRNAs encoded by different viruses showed no sequence conservation, although locations of some of the miRNAs were conserved within the repeat regions of the genomes. However, some of the virus-encoded miRNAs showed significant sequence homology with host miRNAs demonstrating their ability to serve as functional orthologs. For example, mdv1-miR-M4-5p, a functional ortholog of gga-miR-155, is critical for the oncogenicity of Marek’s disease virus. Additionally, we also describe the potential association of the recently described avian leukosis virus subgroup J encoded E (XSR) miRNA in the induction of myeloid tumors in certain genetically-distinct chicken lines. In this review, we describe the advances in our understanding on the role of virus-encoded miRNAs in avian diseases.

## 1. Introduction

MicroRNAs (miRNAs) are ~22-nucleotide small RNA molecules that profoundly affect gene expression by directing repressive protein complexes to the untranslated region (UTR) of target messenger RNA (mRNA) transcripts. Since its first discovery in *C.* *elegans* [1], identification of miRNAs, some of which are evolutionarily conserved [2,3,4], has continued at a fast pace. Today, we know that miRNAs do function as master regulators of gene expression in many species including plants, worms, flies, mammals, as well as in a number of viruses. Out of over 24,000 miRNAs identified until now, 295 are encoded by viruses (miRBase v.20). DNA viruses encode most of the virus-encoded miRNAs, with members of the family *Herpesviridae* accounting for the vast majority demonstrating the significance of miRNA-mediated gene regulation in the biology of herpesvirus infections. Virus-host interactions in herpesviruses are characterized by long-term survival as latent infections in different cell types. This demands sophisticated methods of survival without being detected by the innate and adaptive immune mechanisms of the host. Herpesviruses achieve this using a variety of mechanisms through restricted gene expression, epigenetic control of viral/host gene expression and translational control [5,6]. The small size of the miRNAs, combined with their ability for specific repression of the expression of multiple transcript targets, make them ideal tools for herpesviruses to reshape the gene expression in an infected cell to favor viral replication. Hence it is not surprising that herpesviruses currently account for 95% of virus-encoded miRNAs identified to date. The number of miRNAs encoded by different herpesviruses varies from as few as 3 miRNAs in herpes B virus to 68 in Rhesus lymphocryptovirus (miRBase v.20). In addition to herpesviruses, miRNAs are also encoded by other virus families [7,8] including the recent report of miRNAs encoded by the human Torque Teno Virus [9]. Although retroviruses have not been widely documented to exploit the miRNA pathway [10], recent demonstration of a conserved cluster of RNA polymerase III (pol III)-transcribed miRNAs from the bovine leukemia virus (BLV) genome [11,12] and E (XSR) miRNA encoded by avian leukosis virus subgroup J (ALV-J) using the canonical miRNA biogenesis pathway [13] showed that retroviruses also exploit the miRNA pathway.

## 2. MiRNA-Encoding Avian Viruses and Associated Diseases

Avian herpesviruses are a major group of pathogens associated with a number of diseases in different species of poultry. All of the pathogenic members of avian herpesviruses belong to the same subfamily *Alphaherpesvirinae*. These include the infectious laryngotracheitis virus (ILTV, Gallid herpesvirus) in the *Iltovirus* genus that induces laryngotracheitis, a contagious viral respiratory tract infection that results in high mortality and severe losses in egg production in infected poultry flocks. Vaccination with live attenuated vaccines is used for the control of this disease, although the safety of vaccine strains has been questionable [14]. *Mardivirus* genus consists of the pathogenic Marek’s disease virus-1 (MDV-1, Gallid herpesvirus 2), attenuated Marek’s disease virus-2 (MDV-2, Gallid herpesvirus 3) and the antigenically related herpesvirus of turkey (HVT, Meleagrid herpesvirus 1). MDV-1 is further grouped into different pathotypes referred to as virulent (vMDV), very virulent MDV (vvMDV) and very virulent plus (vv+MDV) based on their pathogenicity [11]. Marek’s disease (MD), caused by MDV-1, is a lymphoproliferative disease of chickens characterized by rapid-onset lymphomas in multiple organs, and infiltration into peripheral nerves causing paralysis. MD is widespread in the poultry population around the world with estimated annual economic losses of US $2,000 million [15]. Although controlled by the use of live attenuated MDV-1 strains and antigenically-related, but non-pathogenic MDV-2 and HVT vaccines [16], there is evidence of continued evolution of the virus towards greater virulence, challenging the sustainability of the vaccination strategy [17,18]. Additionally, avian herpesviruses include other unassigned viruses such as the duck enteritis virus (DEV) that induces acute disease in waterfowl species with high mortality [19,20]. 

Avian leukosis viruses (ALVs), which belong to the genus *Alpharetrovirus* of the *Retroviridae* family, cause neoplastic diseases and other reproduction problems in the poultry industry worldwide. The ALVs can be classified as endogenous (ALV-E) or exogenous viruses according to their mode of transmission. Exogenous ALVs from chickens have been further divided into different subgroups (A, B, C, D, and J) on the basis of their host range, viral envelope interference, and cross-neutralization patterns [21]. Since it was first described in the UK in the late 1980s [22], ALV-J has been primarily associated with myeloid leukosis in meat-type chickens and caused more serious damage than any other subgroup worldwide. ALV-induced disease is still widespread in poultry population in China, where it continues to cause huge economic losses [23].

## 3. Identification of miRNAs Encoded by Avian Viruses

**Figure 1:**
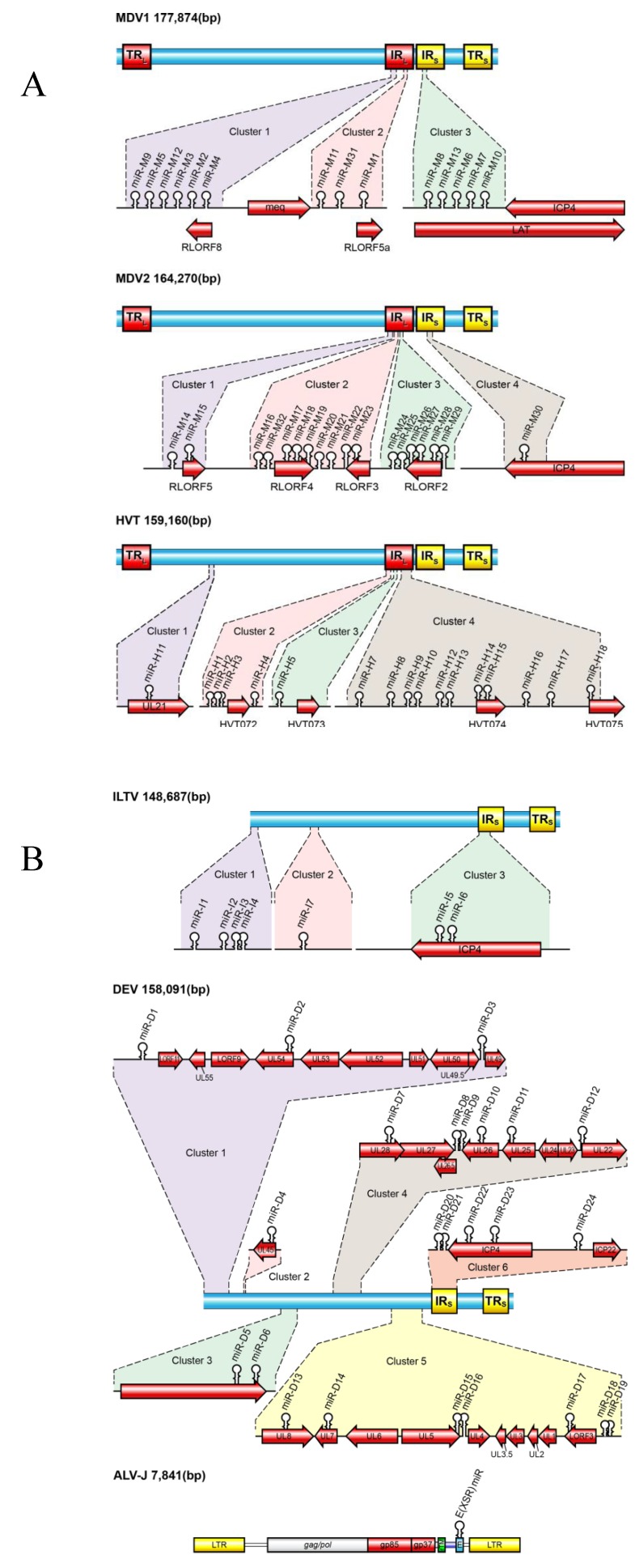
Diagrammatic representation of the viral genomes showing the positions of miRNAs of Marek’s disease virus-1 (MDV1), Marek’s disease virus-2 (MDV2), herpesvirus of turkey (HVT) (**A**) and infectious laryngotracheitis virus (ILTV), duck enteritis virus (DEV) and avian leukosis virus subgroup J (ALV-J) (**B**). Position and orientation of selected transcripts are shown. The genome size (base pairs) of viruses is shown on the top-left corner.

Most viral miRNAs had initially been identified by a protocol involving RNA size fractionation, ligation of linkers, reverse transcription, concatamerization, and Sanger sequencing [7]*.* The computational approaches that rely on commonalities in the predicated secondary structures of pre-miRNAs to identify miRNA-encoding loci specifically in viral genome have also been developed. With the advent of massively parallel sequencing technologies it is now possible to explore libraries of the cloned small RNAs with a higher degree of reliability and unprecedented depth. We and others have reported the identification of miRNAs from a number of avian herpesviruses including 14 miRNAs (26 mature sequences) from MDV-1 [24,25], 18 (36 mature sequences) from MDV-2 [26,27], 17 (28 mature sequences) from HVT [27,28], 7 (10 mature sequences) from ILTV [27,29], 24 (33 mature sequences) from DEV [30], and 1 (2 mature sequences) from ALV-J [13] (Figure 1 and Table 1).

**Table 1:** Avian virus encoded miRNAs and proposed functions highlighted in this review.

Virus	pre-miRs	mature miRs	miRNAs with proposed function	Target	Proposed function
MDV-1	14	26	mdv1-mir-M3	Smad2	Anti-apoptotic [31].
			mdv1-mir-M4-5p	Pu.1, CEBPβ, HIVEP2, BCL2L13, PDCD6, GPM6B, RREB1, c-Myb, MAP3K7IP2	Mimics cellular mir-155 [32,33,34].
			mdv1-mir-M4-3p	UL28	Prevent lytic replication/promote latency [33]
				UL32	Prevent lytic replication/promote latency [33]
			mdv1-mir-M7-5p	ICP4 and ICP27	Establish and/or maintain latency [ 35]
			mdv1-mir-M2	R-LORF8	Lymphocyte growth [34]
			mdv1-mir-M3		
			mdv1-mir-M4		
			mdv1-mir-M12		
MDV-2	18	36	mdv2-mir-M29	R-LORF2	Lymphocyte growth [34]
			mdv2-mir-M28		
			mdv2-mir-M27		
			mdv2-mir-M26		
			mdv2-mir-M25		
			mdv2-mir-M24		
HVT	17	28			
ILTV	7	10	iltv-mir-I5	ICP4	Establish and/or maintain latency [36]
DEV	24	33			
ALV-J	1	2	E(XSR)miR		Potential role in oncogenesis

### 3.1. MDV-1 miRNAs

The first MDV-1-encoded miRNAs were identified in 2006 using high throughput sequencing of small RNA libraries made from chicken embryo fibroblast (CEF) infected with the highly virulent RB1B strain of MDV-1 [24]. More MDV-1 miRNAs were discovered subsequently by analysis of small RNA library of MSB-1, a lymphoblastoid cell line established from an MDV-induced lymphoma of the spleen [25,37]. To date a total of 14 precursor sequences, which produce 26 mature miRNAs, have been identified in the MDV-1 genome (miRBase v.20; Figure 1A). The MDV-1 miRNA coding sequences are clustered into three separate genomic loci: cluster 1 and cluster 2 flank the *Meq* oncogene; cluster 3 lies in the region encoding the LATs [24,25,37] (Figure 1A). All three clusters are located in the inverted repeat regions of the MDV-1 genome. In contrast to the lack of sequence conservation among the different avian herpesviruses, the miRNA sequences are highly conserved among 23 different MDV-1 strains of differing virulence [37,38]. Despite this, the cluster 1 miRNAs are expressed at higher levels in lymphomas produced by vv+MDV than those produced by a vvMDV strain and one polymorphism in the promoter region of these viral miRNAs could be responsible for this differential expression, whereas the LAT-cluster miRNAs expression is equal [37,39], implying that cluster 1 miRNAs may have a more significant role in MD oncogenesis. Indeed, this hypothesis was proved by demonstration that the deletion of the cluster 1 from the viral genome greatly decreased the oncogenicity of the virus [40,41]. mdv1-miR-M4-5p, a member of cluster 1 miRNA and a functional ortholog of gga-miR-155 which plays a major role in lymphoid malignancies and the modulation of immune responses, is the most highly expressed viral miRNA in tumors, representing over 70% of MDV miRNA sequencing reads [37]. This miRNA was shown to play a key role in MDV-1 induced oncogenesis [40,41]. A recent report has shown that the transcription of cluster 1 and 2 miRNAs is driven by a single promoter, prmiRM9M4, corresponding to the 1300-bp immediately upstream from mdv1-miR-M9, under two distinct transcriptional models during different infection phases [39]. Indeed, this promoter has been shown to be active by both active histone marks and DNA hypomethylation during MDV-1 latency [42], confirming its transcriptional activity. A p53-dependent promoter, which has no consensus core promoter element but contains at least two 60-bp tandem repeats harboring a p53-response element, has been found to drive the transcription of LAT-clustered miRNAs [43]. 

Studies have been undertaken to try to correlate a miRNA expression signature with MDV-transformation. The global miRNA expression profiles in seven distinct MDV-transformed cell lines were determined by microarray analysis [44]. These profiles revealed a set of host miRNAs, the expression of which is altered in the MDV-transformed cell lines compared to non-MDV avian viral transformed cell lines or uninfected splenocytes and CD4^+^ T-cells. The host miRNAs downregulated in the MDV-transformed cell lines include miR-155, miR-223, miR-150, miR-451, and miR-26a. Several mammalian homologs of these miRNAs have been linked to cancer development [45], suggesting that the differential expression of these host miRNAs may contribute to MDV pathogenesis. For example, miR-26a is globally down-regulated in a number of avian lymphoma cells, reiterating the highly conserved tumor suppressor function of this miRNA. Functional analysis revealed that miR-26a regulates the expression of interleukin 2 (IL-2) [46]. One established function of IL-2 is in the regulation of T-cell proliferation, which suggests that the decreased expression in miR-26a and the subsequent increase in IL-2 expression could be a conserved mechanism in avian viral transformation. Taken together, analysis of the functional targets of the altered miRNAs would contribute to the understanding of the molecular pathways of MD oncogenicity.

### 3.2. MDV-2 miRNAs

MSB-1 is an MDV-transformed lymphoblastoid cell line co-infected with both MDV-1 strain BC-1 and MDV-2 strain HPRS24 [47]. Seventeen novel MDV-2-specific miRNAs were identified by analysis of the small RNA library from MSB-1 [26]. Out of these, 16 were clustered in a 4.2-kb long repeat region that encodes R-LORF2 to R-LORF5 and are expressed in the same direction suggesting that they may be derived from a common primary transcript. The single miRNA outside the cluster was located in the short repeat region, within the *C*-terminal region of the ICP4 homolog. The identification of miRNA clusters within the repeat regions of MDV-2 demonstrates conservation of the relative genomic positions of miRNA clusters in MDV-1 and MDV-2, despite the lack of any miRNA sequence homology. A subsequent study using high throughput sequencing of small RNAs from MSB-1 cells reported an additional miRNA mdv2-miR-M32 [27]. The unique feature for MDV-2 encoded miRNAs is that all 18 precursors give rise to 2 mature forms, representing both strands of the duplex (miRBase v.20; Figure 1A), resulting in 36 mature MDV-2 miRNAs. Sequencing data from MSB-1 small RNA library showed that MDV-2 miRNAs accounted for 10% and 13% of the sequencing reads in two separate studies [25,27].

### 3.3. HVT miRNAs

HVT-encoded miRNAs were identified from HVT-infected CEF using both high throughput sequencing technology [27] and traditional cloning and sequencing of a small RNA library [28]. These studies identified 17 precursor miRNAs in the HVT genome producing 28 mature miRNAs (miRBase v.20; Figure 1A). The majority of HVT miRNAs are located in the long-repeat regions, with the exception of miR-H11, which is located in the UL region within an intron of UL21, demonstrating some degree of positional conservation with MDV-1 and MDV-2. Ten of the miRNAs were located in a region containing two tandem repeats, and multiple sequence alignment of the miRNA precursors indicated small sequence variations suggesting evolution by duplication. HVT-encoded miRNAs represent the first example of evolution of virus-encoded miRNAs by duplication.

### 3.4. ILTV-miRNAs

A total of seven precursor miRNAs producing 10 mature miRNAs have been identified with the use of deep sequencing of small RNA populations from ILTV-infected chicken embryo kidney (CEK) cells and leghorn male hepatoma (LMH) cell line [27,29] (miRBase v.20; Figure 1B). Four of the miRNAs (iltv-miR-I1-I4) were located at the extreme terminus of the genome and are not associated with any annotated ORFs. The two most highly expressed miRNAs, miR-I5 and miR-I6, are located in the repeat regions within ICP4. The iltv-miR-I7 was mapped in the replication origin (oriL) of the palindrome stem loop sequence. Although the expression of all ILTV miRNAs were confirmed by the end point PCR using small RNA libraries generated from ILTV-infected CEK, only three (iltv-miR-I3, -I5, -I6) have been confirmed by Northern hybridizations.

### 3.5. DEV miRNAs

Small RNA profiles generated by deep sequencing of small RNAs from DEV-infected CEF cultures identified 24 pre-miRNA sequences within the DEV genome producing 33 mature miRNAs [30] (miRBase v.20; Figure 1B). Unlike most *Mardivirus*-encoded miRNAs which are located at the repeat regions, DEV-encoded miRNAs are unique in regards to their genomic positions. The majority of the DEV miRNAs were encoded within the unique long region as six clusters from both the coding and non-coding regions of the 15,809-bp viral genome. The precursors of DEV miR-D18 and miR-D19 overlapped with each other suggesting similarities to miRNA-offset RNAs, although only the dev-miR-D18-3p was functional in reporter assays. Using a computational approach, 12 putative DEV miRNAs have been reported [48], although none of these miRNAs overlapped with the 24 DEV miRNAs described previously [30]. 

### 3.6. ALV-J miRNAs

The sequence of HPRS-103, the ALV-J prototype virus, shows several distinct features, one of which is the presence of a distinct hairpin stem-loop structure called the E (also called XSR) element in the 3' untranslated region. Although the hairpin-like structures of E element suggestive of miRNA precursors, the existence of any mature miRNA has not been demonstrated in ALV-J-infected/transformed cells until the recent published data confirmed the expression of this miRNA [13]. Using deep sequencing approach on one of the ALV-J-transformed cell lines IAH30, a novel small RNA population encoded from within the E (XSR) element, designated as E (XSR) miRNA was identified [13] (Figure 1B). E (XSR) miRNA alone accounted for a quarter of the 1.469 × 10^6^ sequences of the IAH30 “miRNAome”, suggesting that this miRNA has a major role in ALV-J pathogenesis and neoplastic transformation. Although the E element *per* *se* is not absolutely essential for tumor induction by ALV-J, our previous work comparing the oncogenicity of viruses derived from the parental and E element deleted HPRS-103 viruses showed that the E element does contribute to the oncogenicity in certain genetic lines of chicken [49]. Future studies comparing the genomes of these lines could provide insights into the polymorphisms, including those in any potential E (XSR) miRNA target sites that could account for differential susceptibility phenotypes among these lines. 

## 4. Viral Orthologs of Host miRNAs

Virus-encoded miRNAs can be grouped into two classes: orthologs of host miRNAs and viral specific miRNAs. Similar to some virus-encoded regulatory proteins, a subset of viral miRNAs have evolved to mimic host effectors. The “seed” region of a miRNA (~nucleotides 2–8 at the 5' end) plays an especially important role in directing RISC to mRNA targets. It is estimated that ~60% of regulation by a particular miRNA is due to binding with perfect seed complementary to the target transcript [50]. A fraction of virus-encoded miRNAs share seeds with host miRNAs and at least three viruses: Kaposi’s Sarcoma-associated Herpesvirus (KSHV), MDV-1, and BLV have been shown to negatively regulate transcripts via the same target docking sites as their counterpart host miRNAs [32,51,52]. Mimicking a host miRNA allows a viral miRNA to potentially regulate hundreds of transcripts that have evolved target sites for a particular host miRNA. Such regulatory networks evolve to effect specific functions, for example inhibiting apoptosis.

MDV-1 and KSHV express two distinct miRNAs that function as an ortholog of miR-155, a conserved cellular miRNA that is highly expressed in activated lymphoid and myeloid cells and that is required for the rapid expansion of B and T cells after antigenic stimulation [32,37,51,52]. Interestingly, in both MDV-1 and KSHV-induced tumors, there is down-regulation of endogenous levels of miR-155 [44,51,53], although the mechanisms for such down-regulation is not fully understood. There has been a number of studies showing that miR-155 has direct role of oncogenesis [54,55] and induction of cancer [56,57]. Furthermore, overexpression of miR-155 has been shown to be associated with lymphocyte transformation by EBV [58] and reticuloendotheliosis virus strain T [59]. It is therefore striking that MDV-1 induces rapid-onset T-cell lymphomas with transformed cells expressing high levels of mdv1-miR-M4-5p [32,37]. Moreover, deletion or seed region mutagenesis of mdv1-miR-M4-5p greatly reduces lymphoma induction in infected birds showing the importance of this single miRNA in the induction of tumors [40,41]. Introduction of chicken miR-155 into the miR-M4-deleted MDV-1 virus at least partly restored transformation ability, providing the first demonstration that viral miRNAs can play a key role in enhancing the oncogenic potential of a herpesvirus *in vivo*. However, deletion of mdv1-miR-M4 from vvMDV strain GX0101 genome significantly decreased but didn’t totally abolish its oncogenicity [41]. This report, coupled with the finding that insertion of mdv1-miR-M4 into HVT genome failed to induce tumors [38], suggested that other factors are also needed for viral transformation. 

One of the HVT-encoded microRNAs, miR-H14-3p, showed close sequence identity to the chicken gga-miR-221 with perfect match of the 21/23 nucleotides including identical sequence of the seed region, suggesting that it is a virus-encoded ortholog [27]. Compared to other known viral miRNA orthologs, where the sequences are similar only in the seed region, miR-H14-3p was almost identical to the full length mature miRNA, strongly suggesting that hvt-miR-H14-3p is most likely to have been acquired from the host genome. This is the first virus-encoded miRNA that shows such close and extended sequence identity with a host miRNA. Further evidence for this comes from the demonstration of partial sequence conservation between the downstream flanking region of hvt-miR-H14-3p in the HVT genome and the gga-miR-221 locus on chromosome 1 of the chicken genome. If this is true, hvt-miR-H14-3p appears to be the first example of a mature miRNA “pirated” by the virus from the host, despite of the fact that herpesviruses have frequently pirated host cell genes and subverted them to their own purposes. Interestingly, miR-221 targets the cyclin-dependent kinase inhibitor 1B (p27, Kip1), a regulator of the cell cycle G1 to S phase transition. Similar to the miR-221-mediated repression of p27 in cancer progression [60,61], MDV-1-induced tumorigenesis may also involve a similar mechanism [62]. The expression of a miR-221 ortholog and down-regulation of p27 could move the cell cycle to the S phase in order to support replication of the viral genome as well as to increase growth of infected cells for additional viral production [38]. There was also sequence homology between mdv1-miR-M31 and miR-221 in the seed region [37], but is limited to the nucleotide positions 2–7, corresponding to the minimal miRNA seed region. Seed sequence homology has also been observed between mdv2-miR-M21 and miR-29, which is known to function both as an oncogene and a tumor suppressor depending on the context [63]. BLV-encoded miRNA, blv-miR-B4, has been shown to be a functional ortholog of host miR-29 [11]. Furthermore, EBV-encoded miR-BART1-3p and RLCV-encoded miR-rL1-6-3p also share seed sequence with miR-29. Thus, a picture is emerging wherein virus-encoded miRNAs show evidence of targeting host pathways [64]. 

It is estimated that ~8% of currently annotated avian virus-encoded miRNAs could mimic host miRNAs by possessing identical heptameric seed sequence [7]. However, some seed matches between host and viral miRNAs could arise by chance as it is not clear whether all of the currently annotated viral or host miRNAs are bona fide miRNAs based on low abundance, untested biogenesis, and unknown functional relevance. Therefore, it seems likely that only a minority of virus-encoded miRNAs truly mimics host miRNAs, and further validation is required for any proposed orthologs.

## 5. Target Identification of Avian Herpesvirus miRNAs

Although nearly 300 virus-encoded miRNAs are known, an in depth functional understanding is lacking for most. However, it is clear that virus-encoded miRNAs can target both viral and cellular transcripts and recent study analyzing the mRNA targetome has confirmed this [34]. The best characterized viral miRNA functions include subtle roles in supporting viral replication by promoting cell survival, proliferation, and/or differentiation; evading the immune response; and regulating the latent-lytic switch. Notably, all of these functions are particularly important during persistent infections. Modulation of the host cell environment is achieved by multiple and partly redundant mechanisms as viral miRNAs and proteins work synergistically to promote a cellular environment favorable to the completion of the viral life cycle. A number of targets of avian herpesvirus-encoded miRNAs have been identified (Table 1).

## 6. Viral Targets of Viral miRNAs

Identifying viral targets of viral miRNAs is more straightforward compared to identification of cellular targets as viral genomes encode fewer candidate mRNAs. Known examples of viral mRNA targets include transcripts that are transcribed antisense to the viral miRNA precursor and transcripts with imperfect matches. Perhaps the former one is the most straightforward examples of determining viral miRNA function as the miRNA would be predicted to result in a siRNA-like cleavage of the target mRNA if both are co-expressed. For example, iltv-miR-I5 that lies antisense to ICP4 directs cleavage of ICP4 mRNA [36]. ICP4 is an immediate early viral transactivator with a key role in the induction of lytic replication. The targeting of ICP4 by viral miRNAs is thought to mediate entry into latency and render the latent state more robust [65]. In addition to iltv-miR-I5, iltv-miR-I6 also maps antisense to the ICP4 gene. However, repression of luciferase activity observed for iltv-miR-I6 was not significant [36]. The blockage of accessibility to the binding region has been proposed by performing *in silico* folding of RNA containing the targets for iltv-miR-I5 and iltv-miR-I6. This is consistent with the finding that target RNA folding is a key determinant of the efficacy of designed siRNAs [66,67]. Sequences transcribed antisense to known miRNA stem-loop structures may have an increased propensity to fold into stem-loop structures themselves. This strategy could allow a virus to encode miRNAs antisense to mRNA transcripts without altering viral gene expression from transcripts lying on the other strand. Although MDV-1, MDV-2 and DEV also encode miRNAs which are antisense to certain viral transcripts [24,25,26,27,28], possible regulatory relationships between miRNAs and their antisense mRNA transcripts need to be individually verified.

As reported above, mdv1-miR-M4-5p is a functional ortholog of cellular miR-155. Apart from several cellular targets shared with miR-155, mdv1-miR-M4-5p and mdv1-miR-M4-3p have been shown to inhibit the production of the viral UL28 and UL32 proteins respectively, thus providing the first evidence of late viral gene targeting by herpesviral miRNA and making it the first avian herpesvirus miRNA known to target both viral and cellular mRNAs [33]. Both target sequences are located in the coding region rather than 3'UTR. UL28 and UL32 are homologous to human herpesvirus 1 (HHV-1) proteins required for the cleavage and packaging of virion DNA. UL28 and UL32 homologs have been found in all subfamilies of mammalian and avian herpesviruses. However, the role of UL28 and UL32 in MDV-1 packaging has yet to be demonstrated. It appears likely that UL28 and UL32 are involved in the later stages of MDV replication, thus it is possible that mdv1-miR-M4 helps to maintain MDV-1 latency by down-regulating the production of UL28 and UL32 and impairing late MDV morphogenesis and reactivation. 

Two MDV immediate-early (IE) genes, ICP4 and ICP27, have been identified as potential targets for mdv1-miR-M7-5p, a member of the MDV-1 cluster 3, by bioinformatics prediction and subsequent experimental validation [35]. Particularly, there is a negative correlation between the decreased expression of mdv1-miR-M7-5p and an increase in ICP27 expression during virus reactivation. This is consistent with the early finding that miR-M7-5p is highly expressed in MSB-1 cells but at extremely low levels in MDV-infected CEF [25]. By targeting two IE genes, MDV miRNAs produced from LAT transcripts may contribute to the latency. These findings further support the view that herpesvirus miRNAs play a key role in controlling the switch between lytic and latent infection [68,69]. 

## 7. Cellular Targets of Viral miRNAs

Viruses such as herpesviruses with long latency periods need to keep the host cells alive long enough. This time period is greatly extended for viruses that establish latent infection. Thus, prolonging cell survival and evading immune recognition are at least two ways in which viral miRNAs can promote virus replication. As observed for its KSHV-encoded counterpart, several transcription factors are potentially shared targets for both miR-155 and mdv1-miR-M4-5p. These include common targets such as Pu.1, CEBPβ, HIVEP2, BCL2L13, PDCD6, MAP3K7IP2, GPM6B, JARID2, WEE1, RAP2A, RREB1 and c-Myb [32,33,34]. These observations provide additional evidence for the impact of miR-155 and its orthologs on pathways regulating lymphocyte activation, differentiation and immune tolerance [7]. 

MDV1-encoded miRNA miR-M3 targets the transcript of host gene Smad2, a critical component in the transforming growth factor β signal pathway and has been shown to suppress drug-induced apoptosis in cell culture [31]. This data suggests that latent/oncogenic viruses may encode miRNAs to directly target cellular factors involved in antiviral processes including apoptosis, thus proactively creating a cellular environment beneficial to viral latency and oncogenesis. Surely, with the advances in high-throughput technologies, we will continue to identify more targets of miRNAs encoded by other avian viruses. Indeed, a recent study analyzing the whole transcriptome demonstrated IL-18 as a target of MDV-1 and MDV-2 miRNAs [34]. Once more targets of these virus-encoded miRNAs are discovered, and an integrated approach for demonstrating the functions and molecular pathways are developed, we should be able to understand the role played by these small and highly effective modulators of gene expression. 

## 8. Conclusions

Recent advances in sequencing technology have led to the identification of a number of miRNAs encoded by avian viruses. Similar to host miRNAs, it will be imperative to determine which of the reported viral miRNAs possess biologically relevant activities. Although the function of most viral miRNAs is lacking, it is clear that viral miRNAs play a key role in virus biology. The virus-encoded miRNAs could contribute significantly towards switching between lytic and latent infections by directly targeting key viral lytic genes or indirectly modulating cellular regulatory pathways, thereby regulating viral pathogenesis *in vivo*. One key question that remains to be answered is whether these miRNAs have a small number of critical targets, or whether multiple, possibly hundreds, of miRNA-target interactions have functional significance.
